# Rapid Diversity Loss of Competing Animal Species in Well-Connected Landscapes

**DOI:** 10.1371/journal.pone.0132383

**Published:** 2015-07-28

**Authors:** Peter Schippers, Lia Hemerik, Johannes M. Baveco, Jana Verboom

**Affiliations:** 1 Dep. Alterra—Biodiversity and Policy, Wageningen University and Research Centre, PO Box 47, NL-6700AA, Wageningen, The Netherlands; 2 Biometris, Department of Mathemical and Statistical methods, Wageningen University, PO Box 16, NL-6700AA, Wageningen, The Netherlands; 3 Dep. Alterra—Environmental risk assessment, Wageningen University and Research Centre, PO Box 47, NL-6700AA, Wageningen, The Netherlands; 4 Dep. Forest ecology and forest management, Wageningen University, PO Box 47, NL-6700AA, Wageningen, The Netherlands; University of Sydney, AUSTRALIA

## Abstract

Population viability of a single species, when evaluated with metapopulation based landscape evaluation tools, always increases when the connectivity of the landscape increases. However, when interactions between species are taken into account, results can differ. We explore this issue using a stochastic spatially explicit meta-community model with 21 competing species in five different competitive settings: (1) weak, coexisting competition, (2) neutral competition, (3) strong, excluding competition, (4) hierarchical competition and (5) random species competition. The species compete in randomly generated landscapes with various fragmentation levels. With this model we study species loss over time. Simulation results show that overall diversity, the species richness in the entire landscape, decreases slowly in fragmented landscapes whereas in well-connected landscapes rapid species losses occur. These results are robust with respect to changing competitive settings, species parameters and spatial configurations. They indicate that optimal landscape configuration for species conservation differs between metapopulation approaches, modelling species separately and meta-community approaches allowing species interactions. The mechanism behind this is that species in well-connected landscapes rapidly outcompete each other. Species that become abundant, by chance or by their completive strength, send out large amounts of dispersers that colonize and take over other patches that are occupied by species that are less abundant. This mechanism causes rapid species loss. In fragmented landscapes the colonization rate is lower, and it is difficult for a new species to establish in an already occupied patch. So, here dominant species cannot easily take over patches occupied by other species and higher diversity is maintained for a longer time. These results suggest that fragmented landscapes have benefits for species conservation previously unrecognized by the landscape ecology and policy community. When species interactions are important, landscapes with a low fragmentation level can be better for species conservation than well-connected landscapes. Moreover, our results indicate that metapopulation based landscape evaluation tools may overestimate the value of connectivity and should be replaced by more realistic meta-community based tools.

## Introduction

**Fig 1:**
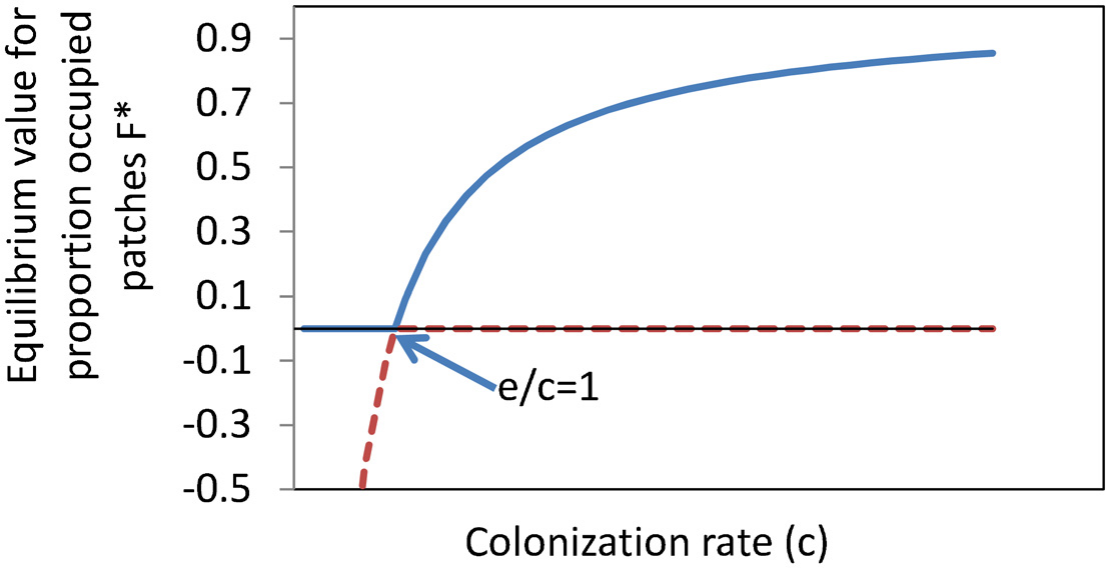
Fraction of occupied patched in a metapopulation based on the Levins model dependents on colonization rate. The dotted red line represents the unstable equilibrium, and the drawn blue line the stable equilibrium. The figure illustrates that there is a connectivity threshold (*c*/*e* = 1) below which long term survival of a metapopulation is impossible.

Models and tools based upon the metapopulation concept play a key role in evaluating optimal habitat configurations with respect to species survival in fragmented landscapes [1–5]. The behavior of a single-species metapopulation is well-understood when considering Levins’ simple metapopulation model [6]. In this model the fraction of occupied patches at equilibrium F* depends on the colonization rate *c* and the extinction rate *e* of the local populations (eq 1 and Fig 1)[6].

$$Ex:\;F*=1-\frac{e}{c}\text{ for}\;c>e$$ (1)

F*=0  ​ ​​forc<e

If the fragmentation of a landscape increases, the colonization rate *c* decreases, and consequently *e/c* increases and the fraction of occupied patches F* decreases; beyond the *e/c* = 1 threshold, F* is zero (Eq 1) meaning that there is no long term species survival possible beyond this fragmentation threshold (Fig 1). So, metapopulation theory predicts that with the decrease of inter-patch connectivity a species is found in fewer patches. When a landscape is too fragmented a population cannot survive at all [6–8]. More complex, e.g. individual-based metapopulation models are all a variation on this theme and share this same behavior [1]. If metapopulation theory is applied to multiple species without interaction, the number of species that survives in the landscape increases with increasing connectivity. This means that analyses done with metapopulation based models and tools always predict that single large patches and less fragmented landscapes are better for species diversity and conservation than more fragmented landscapes [1,9–11].

Model studies incorporating interacting species, however, show more complex outcomes. For example, in an unfragmented and homogeneous area, interspecific competition may cause extinction of the weaker competitor [12,13]. Furthermore, coexistence was found to be possible between a superior disperser and a superior competitor in fragmented landscapes [14–17]. In addition, evidence exists in relation to predator-prey dynamics that spatial segregation can lead to stable coexistence of species in a landscape [18–20]. The fact that in unfragmented landscapes superior competitors and predators are able to exclude weaker species and that spatial segregation might be necessary for coexistence, demonstrates that fragmentation can also be a prerequisite for species richness, which contradicts metapopulation theory based expectations. There are also empirical examples in which isolation is a prerequisite for the coexistence between interacting species. For example, many seabird species build nests on coastal islands and cliffs to protect their offspring from terrestrial predators [20–22]. Another example is the competition between invasive and native species, such as the grey and red squirrel. The grey squirrel is a superior competitor that is capable of driving the red squirrel to extinction [21–23]. In Australia some of the native marsupials only survive on isolated islands where invasive predators and/or competitors cannot interact with them [24]. In Spain butterfly diversity was found to be higher in fragmented landscapes with patches of holm-oak forest [25,26]. These examples show that, on various scales, species might profit from spatial segregation. Here, the reduction in interaction between species caused by isolation seems more important than the negative effects of isolation. So fragmentation creates refugia in space and time allowing species to survive.

In the neutral theory of biodiversity, the species richness at a location is an interplay between immigration, establishment, extinction and speciation on an individual basis [27,28]. This theory assumes a hierarchical structure with a large (meta-community) pool of species, and relatively small local populations. Furthermore, the species interaction is simplified in such a way that different species have equal area requirements. In such a neutral model the local diversity has a steady state solution when the gains of new species from the speciation and immigration process are equal to species losses through extinction [27]. Like the metapopulation theory, neutral models predict that high connectivity enhances the local species richness. However, on larger spatial scales, neutral theory predicts that increased connectivity reduces the diversity level [29].

The preceding sections suggest that meta-community and neutral theory based landscape evaluation models, that take into account species interactions, yield different results and recommendations for biodiversity conservation than metapopulation theory based evaluation models. In today’s conservation policy, however, the metapopulation concept plays a key role in evaluating optimal habitat configurations with respect to species survival in fragmented landscapes [1–5], predicting that with increased connectivity more species will survive. However, this generally ignores species interactions. On the other hand meta-community models are frequently used to study mechanisms for coexistence between species, often predicting coexistence in contrasting species. However these studies are often theoretical, ignoring realistic spatial dimensions and the stochastic nature of the extinction process and are therefore of little use to conservation policy makers at national or regional level. In this paper we aim to bridge the gap between metapopulation and meta-community models. To this end, we extend a metapopulation model METAPOP-Alterra [30] to be able to simulate several competing species in a landscape of randomly distributed patches. Using this model, we simulate a community of multiple species that compete with each other in a Lotka-Volterra like way with different competitive settings: (1) weak, coexisting competition (CC), (2) neutral competition (NC), (3) strong, excluding competition (EC), (4) hierarchical competition (HC) and (5) random species competition (RC). In these settings we evaluate the local and overall species richness at various levels of fragmentation. We specifically ask: (1) what is the effect of landscape fragmentation on the diversity of interacting species and (2) what are the mechanisms determining species richness in a meta-community context.

## Material and Methods

We explore a meta-community of 21 interacting bird species governed by Lotka-Volterra competition [31,32] in a spatially explicit landscape of 100 patches in discrete time, with a time step of one year, incorporating demographic and environmental stochasticity. The demographic parameters used are based on parameters from the middle spotted woodpecker [33] (Table 1), a European cavity breeding forest bird whose main food are insects and seeds and whose habitat is often fragmented by natural patchiness and due to human-induced land-use change. These parameters were derived from field work in European old forests [34–41]. The simulated species could therefore represent a woodland bird community in a landscape with forest patches. The Lotka-Voltera competition indices ruling species interaction are however determined by the competitive setting.

**Table 1:** Description of the parameters, their units and their default values as used in the model.

Description	Symbol	Value	Range*	Unit
***State variable***				
Number of adults in patch k of species i at year *t*	*A*_*k*,*i*_(*t*)	**-**	-	Adults
***Demographic***				
Adult mortality of species *i*	*m*_*i*_	0.3	0.16–0.44	year^-1^
Recruitment of species *i* at zero density	*r*_*i*_	1.8	1.45–2.15	Juvenile per female
Competition coefficient	*α*_*ij*_	varying	0.4–1.6	-
Carrying capacity of patch *k*	*K*_*k*_	20–60	-	Adults
Env. Coef. of variation in recruitment	*CV*_*rec*_	30	-	%
Env. Coef. of variation in survival	*CV*_*sur*_	10	-	%
***Dispersal***				
Probability to disperse adult, all species	*P*_*a*_	0	-	year^-1^
Probability to disperse juvenile at density = 0, all species	*P*_*i*_(0)	0	-	year^-1^
Probability to disperse juvenile at carrying capacity *K*_*k*_, all species	*P*_*i*_(*K*_*k*_)	0.6	0.56–0.84	year^-1^
Radius of the target patch (*l*)	*u*_*l*_	0.28–0.48	-	km
Distance between patches *k* and *l*	*d*_*k*,*l*_	varying	-	km

*Range for random species competition setting

### Local population dynamics

**Fig 2:**
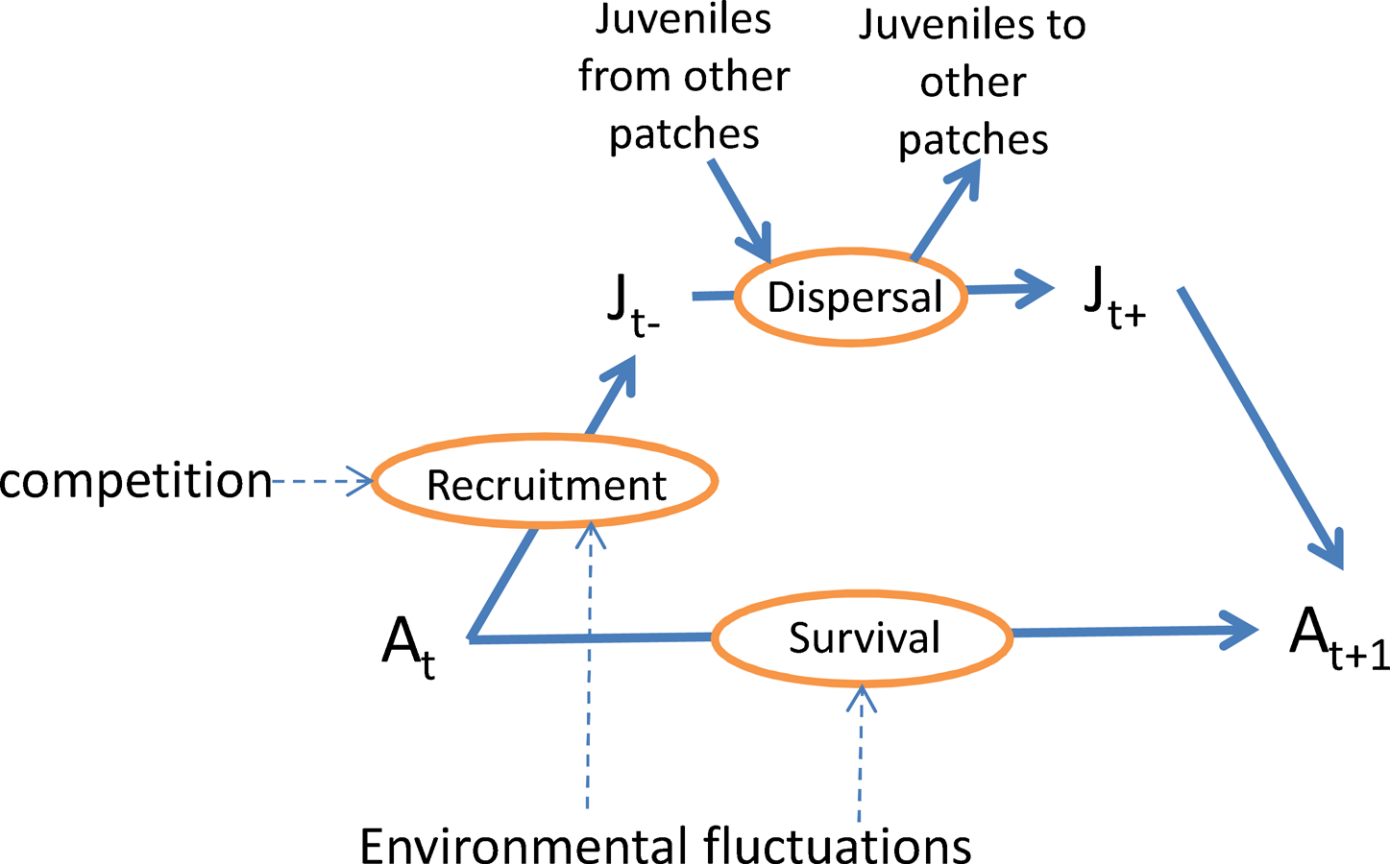
Population model structure of a single bird species in a single patch. A = adult numbers, J = juvenile numbers. Pre-dispersal juvenile numbers are indicated as J_t-_ and after dispersal juvenile numbers as J_t+_. Competition among individuals of different species affects the recruitment. Recruitment and adult survival are affected by environmental fluctuations. A fraction of the juveniles disperses to other patches while dispersers from elsewhere are allowed to settle in the patch. Note that in Eq 1 the juveniles are not considered explicitly because we model pre-breeding adults: from *A*(*t*) to *A*(*t*+1).

Each patch has a carrying capacity (*K*) of on average 20 breeding pairs (namely 40 breeding pairs per km^2^) and the local population dynamics results from the interplay between recruitment (*r*), mortality (*m*), immigration (*I*) and emigration (*E*). A detailed scheme about how the dynamics of the interaction between species is assumed to affect the population sizes is given in Fig 2. The recruitment depends on the density of other species and their conspecifics. The model is fully stochastic with two sexes and discrete population sizes. In a deterministic version of the model the year to year dynamics of the number of adults of species (*i*) in patch (*k*) assessed just before the breeding season (i.e. pre-breeding census) in a patch would be given by Eq 2.

$$Ex:A_{k,i}(t+1)=A_{k,i}(t)+\left(0.5\cdot r_{i}-m_{i}\right)\cdot A_{k,i}(t)\left(1-\frac{\sum_{j=1}^{n}A_{k,j}(t)\cdot \alpha_{ij}}{K_{k}}\right)-E_{k,i}+I_{k,i}$$ (2)

Where *A*_*k*,*i*_(*t*) is the number of adults of species *i* in patch *k* (males and females) at time *t*, *r*_*i*_ is the recruitment of species *i* (year^-1^), this is the number of animals (females and males) that are on average born from a single female and survive the first year at zero density. The factor 0.5 in front of *r*_*i*_ stems from the assumption of a 1:1 sex ratio in newborn animals. The adult mortality of species *i* (year^-1^) is *m*_*i*_ and *α*_*ij*_ is the competition coefficient between species *i* and *j*; the meaning of this parameter is: what resources does species *j* use relative to species *i* with respect to the carrying capacity in the current patch *K*_*k*_, we assume that the carrying capacities of all the species are the same; *E*_*k*,*i*_ is emigration of species *i* from patch *k*, and *I*_*k*,*i*_ is immigration of species *i* into patch *k*. It should be noted that juveniles are not explicitly modelled, because with a time step equal to the age at first reproduction, juveniles born at time *t* are already adult at time *t*+1 (see Fig 2) so the recruitment term combines reproduction and juvenile survival.

### Discrete dynamics

The model is stochastic and uses discrete probability distribution functions with means defined by the deterministic model (eq 2). The model uses a Poisson distribution to assess the results of the recruitment processes. The Poisson distribution is used because it returns a positive discrete number and is therefore a parsimonious way of handling the recruitment in a stochastic way [42–44]. Mortality, on the other hand, is simulated by drawing from a binomial distribution with a survival (= success) probability of (1−*m*). Based on the number Ak,i(t) in a patch a Binomial draw returns the discrete number of surviving individuals in a population [30,43,45]. We also incorporate environmental stochasticity to account for e.g. severe winters, affecting adult survival, and cold springs, affecting recruitment. Because our species have partial or complete niche overlap we assume that all species and all patches are affected by environmental stochasticity in the same way. Environmental stochasticity is modeled by adding Gaussian noise to both recruitment parameter *r*_*i*_ and mortality parameter *m*_*i*_. without any correlation between years or between the events within a year. The expected reproduction is determined by the carrying capacity (interpreted as the number of tree cavities suitable for nesting) and the minimum of both male and female numbers of the same species (assuming monogamy) in a patch. This minimum introduces an Allee effect [46,47], because especially at low densities the sex ratio deviates more strongly from one causing that unpaired (fe)males do not reproduce [48].

### Competitive settings

The competitive interaction in the model is defined by a 21x21 competition matrix of *α*_*ij*_ values that determines interspecific and intraspecific competition (see Eq 1). Intraspecific competition is however identical for all species (*α*_*ij*_ = 1). We distinguish 5 competitive settings for the interspecific competition named after the expected outcome of the competition in well mixed non spatial explicit settings:

**(CC)** Coexisting Competition:*α*_*ij*_ = 0.4 if *i* ≠ *j*, *α*_*ij*_ = 1 if *i* = *j*. In this case, individuals of other species are weaker competitors than individuals of the same species, resulting in coexistence between the species in a deterministic model. Here, we assume partial niche overlap between the species: individuals of other species are in this case less competitive than individuals of the same species. We assume that all species have identical recruitment, mortality, dispersal and carrying capacity mimicking competition in a group of similar forest species having partial niche overlap.
**(NC)** Neutral Competition: all *α*_*ij*_ are 1. Here, all species are identical and are assumed to be equally competitive in acquiring resources assuming complete niche overlap and resource competition. This competitive setting is close to that in the neutral models in which each individual of all species requires the same amount of space [49]. We assume that all species have identical recruitment, mortality, dispersal and carrying capacity, mimicking competition in a group of similar species having complete niche overlap.
**(EC)** Excluding Competition: *α*_*ij*_ = 1.6 if *i*≠j, *α*_*ij*_ = 1 if *i* = *j*. In this competitive scenario, individuals of competing species are stronger competitors compared to individuals of the same species, resulting in fast competitive exclusion in a well-mixed deterministic model. For every species, all other species are regarded as superior in acquiring resources. As a result of this parameterization species that have accidentally high densities due to initial settings or a stochastic event deterministically out-compete species with lower densities in non-spatial explicit settings. Like in the preceding competitive setting we assume that all species in these competitive have identical recruitment, mortality, dispersal and carrying capacity parameters.
**(HC)** Hierarchical Competition: Here, we assume a clear competitive hierarchy between species. Species 1 is superior to all others, species 2 is superior to all but species 1, species 3 is superior to all but species 1 and 2 etcetera. The differences between species are not large, e.g. α_*12*_ *=* 0.98, α_*11*_ *=* 1, α_*21*_ *=* 1.02 (for the exact matrix of all species see S1 Table). We assume again that all species have identical recruitment, mortality, dispersal and carrying capacity parameters.
**(RC)** Random species competition: In the preceding competitive settings we have highly similar species having identical recruitment, survival and dispersal but different competitive setting. Here, we want to mimic a group of woodland bird species that vary in their recruitment, mortality and dispersal having different competitive interactions. To accomplish this we vary the recruitment *r*, adult survival (= 1-*m*), and the dispersal probability P of each species between −20 and +20% of the standard parameters. So we vary recruitment (1.45–2.15), survival of adults (0.56–0.84), and dispersal probability (0.48–0.72). Additionally, the interspecific competition indices *α*_*ij*_ is randomly drawn between 0.4–1.6 while keeping the intraspecific competition indices *α*_*ii*_ to be one for all the species. These randomly drawn parameters are kept constant during the whole simulation.

### Landscape configuration

The landscape consists of 100 randomly distributed patches with randomly drawn sizes between 0.25 and 0.75 km^2^ which correspond to a carrying capacity in these patches of 10–30 breeding pairs. The minimum distance between these randomly distributed patches is 150 meters. In the landscape with the highest connectivity, i.e. the lowest degree of fragmentation, which we indicate as having a fragmentation scale of one, these patches are distributed in a landscape of 14.1 x 14.1 km^2^ yielding a patch density of 0.5 patch per km^2^ and a habitat coverage of the landscape of 25%. To study the effect of habitat fragmentation (or the reverse, connectivity) we increase this scale to higher values. For example, when the scale is ten, the 100 patches have the same size range but are distributed in a landscape of 141x141 km^2^ increasing the average inter-patch distances 10 times (see Eq 3); the average habitat coverage of this landscape is 0.25%. For each simulation a new landscape is created. To illustrate how a well-mixed system performs, we also study the dynamics of the different competitive settings in a single patch of 50 km^2^ having the same carrying capacity as the full population of the fragmented landscape, i.e. 2000 (or 100 patches times the average patch carrying capacity of 20) breeding pairs.

### Dispersal

We assume that only juveniles disperse and that their dispersal probability (*P*_*k*,*i*_) of patch *k* and species *i* scales with local density (*A*_*k*,*i*_ (*t*), Eq 3).

$$Ex:P_{k,i}=\text{min}\left(P_{i}(K_{k}),\frac{P_{i}(K_{k})\cdot A_{k,i}(t)}{K_{k}}\right)$$ (3)

Where *P*_*i*_(*K*_*k*_) is the juvenile dispersal probability of species *i* at carrying capacity *K*_*k*_ of patch *k*.

During dispersal, we assume ballistic (straight-line) movement. Thus the diameter and the distance of a target patch determine the connectivity from the reference patch to the neighbouring patch [4,33]. The probability *P*_*k*→*l*_ that a disperser goes from patch *k* to patch *l* is described in Eq 4.

$$Ex:P_{k\to l}=\frac{2\cdot \text{arcsin}(\text{min}(1,\frac{u_{l}}{d_{k,l}}))}{2\cdot \pi }$$ (4)

Here *u*_*l*_ is the radius of target patch *l*, *d* is the distance between natal patch *k* and target patch *l*. The algorithm accounts for shadowing, i.e. nearby patches intercept the dispersers that otherwise would end up in the patches behind these patches. In the landscape, all patches are connected to each other according to this principle. Note that the probabilities do not add up to one as some dispersers do not encounter a patch and are lost (dispersal mortality). For more model details see S1 File.

### Simulations

The simulations are initialized with a single breeding pair of each species in every patch and run for 1000 or 3000 years at various fragmentation scales ranging from 1 to 100. The species richness of the whole population and the average number of species in a single patch are sampled every year. We perform 30 simulations in each of the settings, so the results in a single setting are averages of these 30 simulations. To avoid bias due to a particular generated random landscape, each simulation takes place in a different randomly generated landscape having the same patch number, size distribution and scale. We perform these simulations for all five competitive settings.

To test whether our results are sufficiently robust to model assumptions, we tested the generality of our results by performing 9 extra sets of simulations by varying: initialization, carrying capacity, patch size range, reproduction, dispersal, environmental stochasticity and competition indices (see ESM other analysis and S2 Table).

## Results

### Single simulations

**Fig 3:**
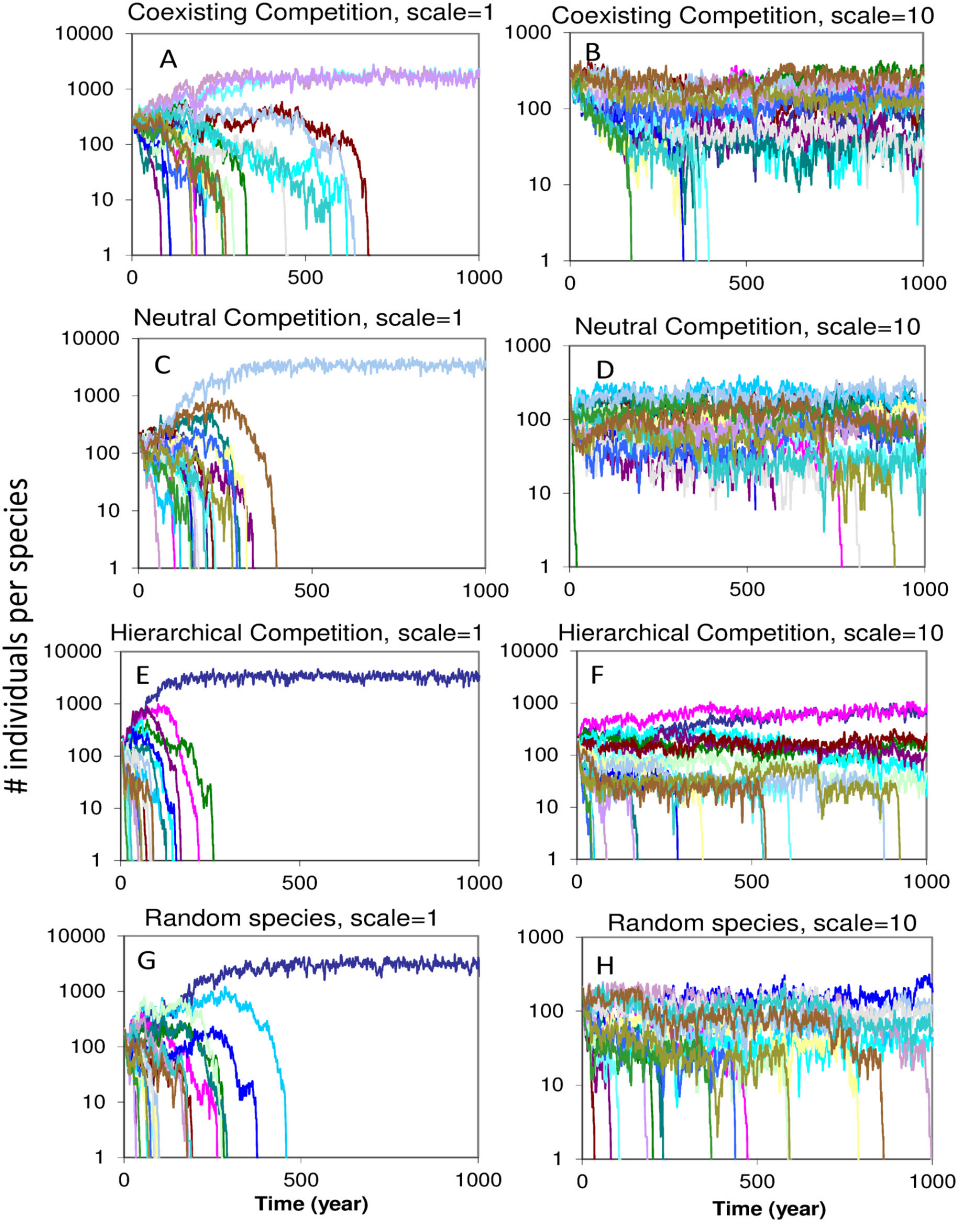
Single representative simulations of the meta-community dynamics of 21 bird species in contrasting competitive settings and inter-patch connectivity (scale). Each graph depicts a single representative simulation. Different lines and colors represent the population level of different competing species. Left column (A, C, E, G) have low inter-patch distances and high connectivity, scale = 1. Right column (B, D, F, H) have 10 times as large inter-patch distances and low connectivity, scale = 10. Competitive setting: (A, B) coexisting competition, (C, D) neutral competition, (E, F) hierarchical competition, (G, H) random species competition. The figure illustrates that at high connectivity we get faster extinction of species in these competitive settings.

Fig 3 shows the results of ten representative individual simulations over time at contrasting competitive settings and at two fragmentation levels, well connected (scale = 1) and fragmented (scale = 10). In the high fragmentation case we see that more species have survived after a thousand years whereas in well-connected landscapes only one or two species have survived. In the hierarchical competitive setting the most competitive species (blue = strongest and pink = second strongest) are the species that are most dominant in the simulations. Here, at scale one, the strongest competitors outcompete all others in 250 years, but at high fragmentation levels (scale 10) 7 out of the 21 species survive until 1000 years. Again, both species with strong competitive ability are also dominant in these simulations.

### Diversity loss in time per fragmentation scale

Next, we systematically explored the temporal dynamics for different landscapes, and for the five different competitive interactions. We monitored species richness (number of species) over time at six spatial scales: single large patch (all habitat merged, *K* = 2000) and 100 patches at scales of 1, 1.6, 4, 13.6 and 103. In contrast with the preceding section the results are the average values from 30 simulations.

**Fig 4:**
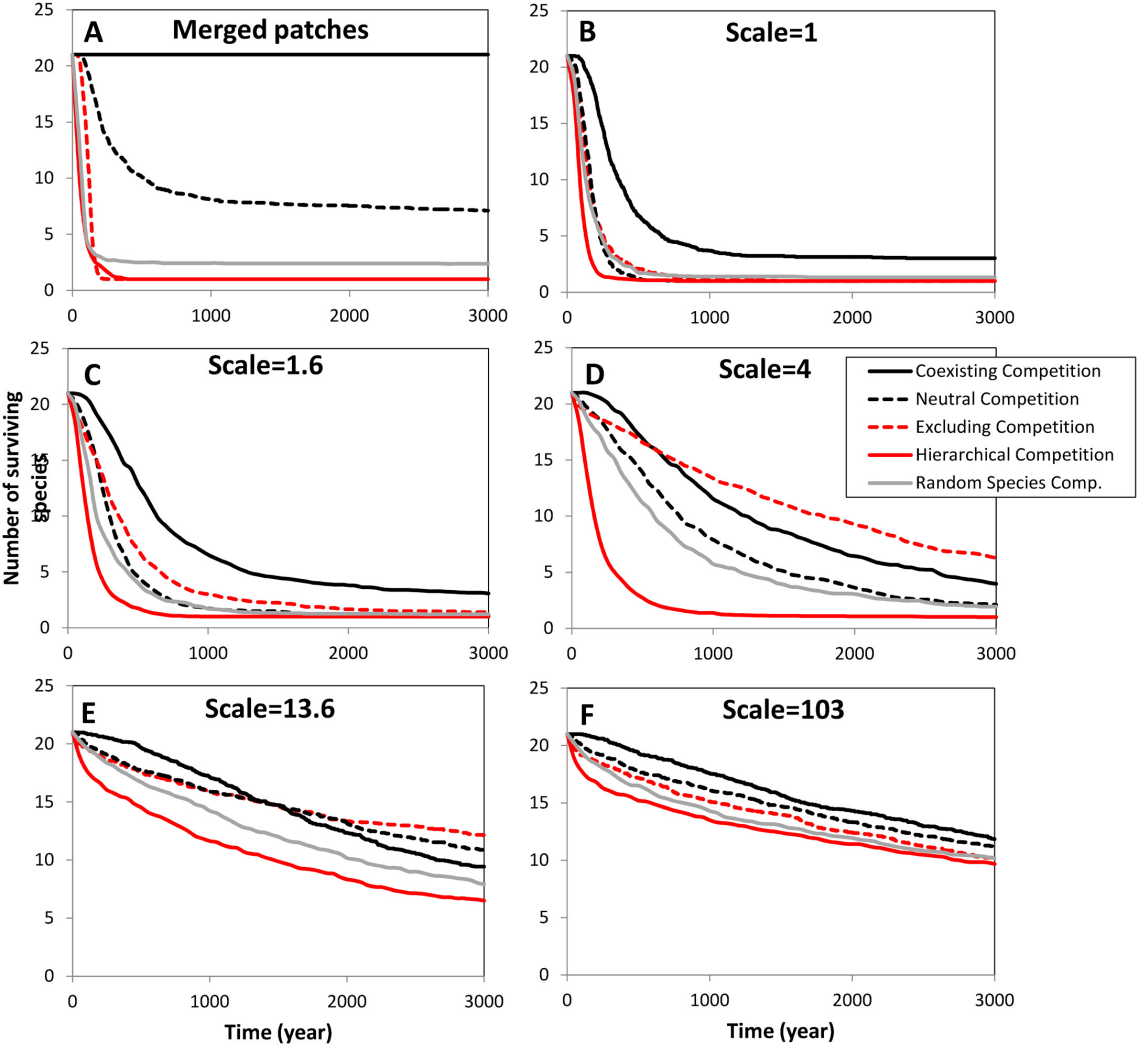
Number of surviving species out of 21 interacting species over time in a meta-community model at different fragmentation levels (scale) and competitive settings. The landscape consists of 100 randomly distributed habitat patches of different sizes (B-F) or a merged patch with equal area (A) at 6 connectivity levels (scales). Merged patch: all habitat of the 100 patches is combined into one single large patch of 50 km^2^, scale = 1: the patch density of 100 patches is on average 0.5 patches km^-2^ in a landscape of 14.1 x 14.1 km^2^. When the scale = 4, the 100 patches having the same size are randomly distributed in an area of 4*14.1 x 4*14.1 km^2^. Different lines represent the number of surviving species at different competitive settings.

In the merged setting, as expected, the coexisting competitive setting (CC) does not show any species loss (Fig 4A), The species richness in neutral settings (NC) decreases quickly between 50 and 500 years until a level of 10 species but, subsequently, the species richness declines slowly having a mean diversity of 7.5 species after 3000 years. The random competitive setting shows a fast decline but stabilizes at 2.4 after 200 years, both excluding and hierarchical competitive settings show a rapid decline yielding only one surviving species after 250 years. Fig 4B describes the species richness in a well-connected landscape which is subdivided into 100 patches. Between 0 and 1000 years, the coexisting competitive setting (CC) declines rapidly from 21 to 3 species. After 1000 year the species richness in this setting, however, no longer declines. The diversity of the other settings also declines strongly, reaching a level of close to one species after 500 years. By increasing the fragmentation scale from 1 to 1.6 we see that the loss of species richness in all competitive settings is slower (Fig 4C). Increasing the fragmentation further to scale = 4 (Fig 4D) the loss of species richness is even slower than in the previous scale, the lowest diversity loss is in the excluding competitive setting. At a fragmentation scale of 13.6 and 103 the species decay is even weaker (Fig 4E and 4F). At a scale of 103, the performance between the competitive settings is roughly equal, yielding a final species richness between 10 and 13 species after 3000 years.

### Effects of fragmentation on species richness at certain points in time (time slices)

**Fig 5:**
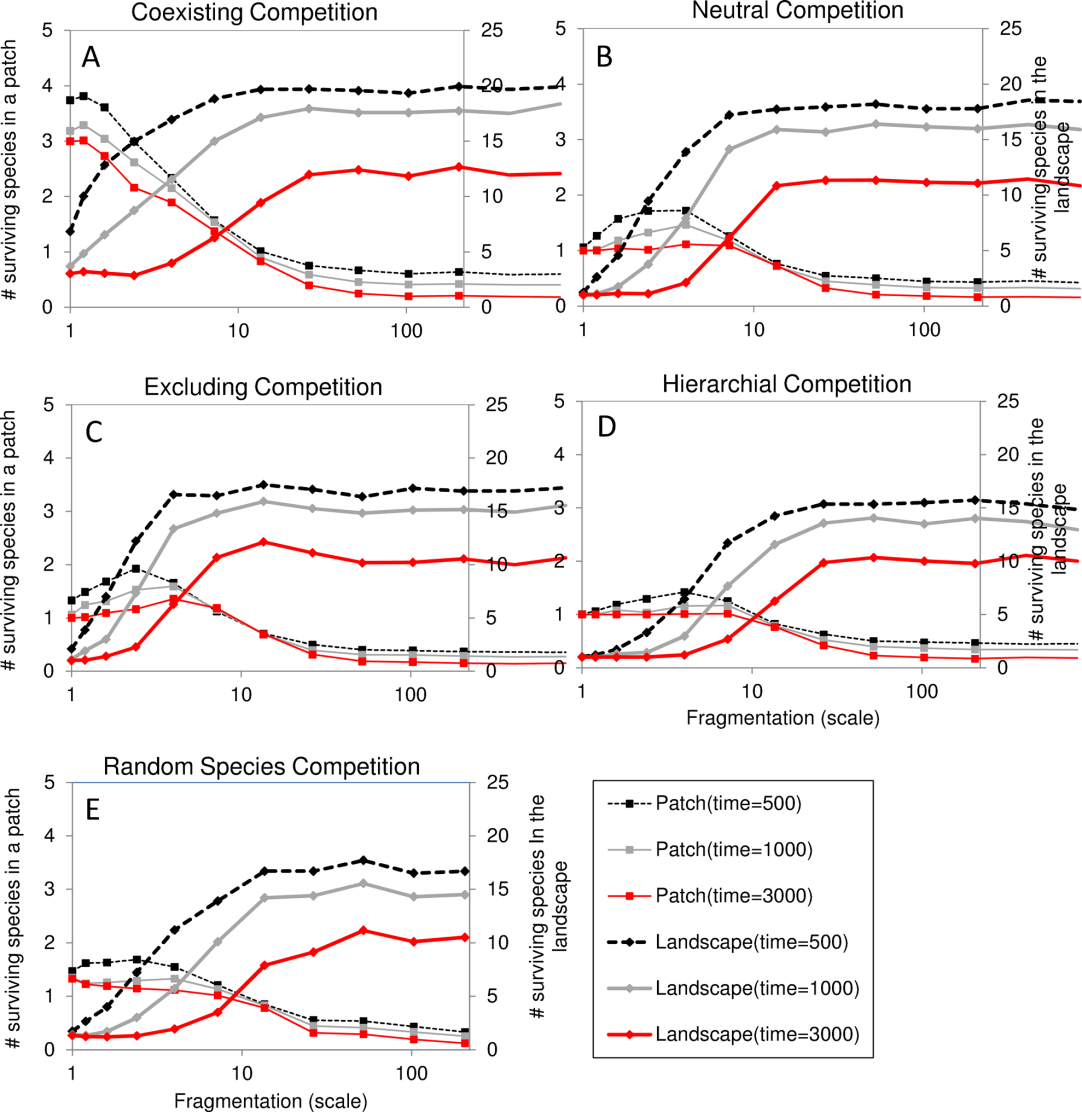
The effect of fragmentation (scale) on the number of surviving species out of 21 under the reference conditions at five competitive settings (A-E). Different lines indicate the amount of surviving species out of 21 at different time intervals (*t* in years) since the start of the simulation. Bold lines indicate the diversity in the whole landscape and thin lines indicate the average patch diversity. Every point represents the average of 30 simulations. The landscape configuration consists of 100 patches that vary in size and are distributed at random, the average patch carrying capacity is 20 breeding pairs. The figure illustrates that the metapopulation diversity is highest at high fragmentation scales in all competitive settings whereas patch diversity is highest at intermediate fragmentation levels.

Next we analyzed the effects of fragmentation scale on the species richness after 500, 1000 and 3000 years. In all competitive settings (Fig 5) the species richness on a landscape level increases strongly with isolation between scales 1 and 10 after which it stabilizes suggesting that high fragmentation levels support species richness. However, this seems to be transient behavior, as diversity decreases with time in all competitive settings (M3000 < M1000 < M500). On the patch level, however, the diversity is much lower as 17–20 out of 21 species are lost in the average patch at low fragmentation, and the number of species drops below one species per patch for high fragmentation. This means that some patches in the landscape are not occupied. The patch level diversity is highest in the coexisting competitive setting: four species at low fragmentation and decreasing to values below one for high fragmentation landscapes. In the neutral (NC), excluding (EC), hierarchical (HC) and random species (RS) competitive settings the patch diversity level is low at low fragmentation (between 1 and 2 species per patch). However, diversity increases slightly for intermediate fragmentation levels before declining to values below one. These values below one at high fragmentation levels originate from the fact that a fraction of the patches is empty under these conditions.

### Other settings tested

To test the generality of our results we also performed nine extra sets of simulations to evaluate various model assumptions: initialization, carrying capacity, patch size range, reproduction, dispersal, environmental stochasticity and random competition indices (S2 Table).

The species richness, which was evaluated after 1000 years was in all cases much higher in the fragmented landscapes compared to the well-connected landscapes. However, when patches have a high carrying capacity, in the coexistence competitive setting (CC) we found a relatively high species richness of eight under well-connected conditions. Nonetheless, even here, the diversity levels are still higher in more fragmented landscapes.

## Discussion

### Main results

We have studied the population viability of a meta-community of 21 competing species, in five competitive settings and at multiple landscape fragmentation levels. According to the single-species metapopulation theory we would expect a higher species survival in well-connected landscapes. We found, in contrast to the metapopulation theory, that species richness at the landscape level was always highest at higher levels of fragmentation, regardless of the form of competition chosen. In the standard simulations, after 3000 years, the species richness was still more than four times higher at higher fragmentation levels compared to low fragmentation levels. In addition, the patch diversity often remained higher at intermediate fragmentation levels. However, the advantage was typically in the order of 50% and this mostly occurred at lower fragmentation levels compared to the maximum diversity at the landscape level. These results were valid in all five competitive settings including the random species competition and were robust to changes in parameter settings and spatial configurations (see Figs 3, 4 and 5 and S2 Table). Nevertheless, over the very long run we expect extinction of all species in the highly fragmented landscapes due to classical metapopulation dynamics (crossing the threshold for metapopulation viability due to a too low colonization rate: *c<e*). All-in-all these results suggest that, if competitive interactions between species are taken into account, spatial segregation supports species richness on the medium long run of 500–3000 years.

### Rapid species loss

Rapid species loss occurs in well-connected configurations, because species that accidentally become dominant send out larger numbers of dispersers that colonize other patches and take over the local populations in these patches. These species are likely to outcompete other species in the patch. The “conquered” patch also sends out dispersers of the already abundant species helping to colonize more patches. Additionally, in well-connected landscapes dispersing juveniles have a higher probability to arrive in patches. This leads to higher population levels in patches. At higher population levels that are closer to carrying capacity we expect a more intense competition and a faster competitive exclusion [50]. This competitive exclusion process causes a rapid shake out of accidentally less abundant species in well-connected landscapes (Fig 3). Over time this leads to a species poor meta-community with one or a few species. In fragmented landscapes this cannot occur because the colonization rates are low, due to smaller patch-to-patch dispersal flows. The colonization process is also hampered by Allee and founder effects [51]. The Allee effects in our stochastic model are generated by the increased probability that sex ratios deviate from one at low densities [30,48]. Additionally, the presence of established competitors inhibits successful colonization because the new colonizer arriving with low numbers experiences competition from them. Both processes induce a founder effect [51], where species that accidently become dominant in a patch prevent others from colonizing. So in a fragmented condition the patches are, in fact, poorly connected and due to stochastic processes multiple populations in a single patch are thinned to a single population per patch. These patch populations have a certain survival time (on average 1*/e*) but without sufficient immigration they are eventually doomed to local extinction. However, close to the metapopulation extinction threshold the time to overall extinction due to these local extinctions can be very long [52] and this process is much slower than the extinctions due to the competitive exclusion process, which involves the monopolization of all patches by a single or a few dominant species in well-connected environments. Our research thus suggests that the competitive exclusion process is much more important than extinction by metapopulation processes when niche overlap is an important factor.

Our results therefore indicate that the rate of diversity loss is affected by two counteracting forces affected by the connectivity of the landscape. With metapopulation-based extinction probability increases due to fragmentation because patch colonization *c* becomes lower and eventually even lower than the patch extinction *e*. Conversely, there is the “competitive exclusion” process which is decreasing with fragmentation because, without spatial segregation, dominant species wipe out the other species. One way of balancing these two processes is by varying the carrying capacity of the patch because this affects population extinction in a patch. However, in simulations where the carrying capacity is reduced and the metapopulation component of extinction is thus increased, the populations still do not profit from this higher connectivity (ESM table 2). This indicates that the competitive exclusion process is also in this case much stronger than the extinction driven by the metapopulation dynamics.

### Competitive settings

We would have expected that species with the coexisting competitive setting (CC), with coexisting species in a deterministic model, would profit from high connectivity in fragmented landscapes because a well-connected landscape is thought to be similar to a single large patch. There was, however, a marked contrast between the single large patch case in which all species coexist and the well-connected multiple patch case in which a rapid species decline stabilizes into a situation with about three coexisting species. At higher levels of fragmentation at least 12 species survive over 3000 years. Thus, even competitive interactions that result in stable coexistence profit from spatial segregation. This is because there is still a 40% niche overlap between these species meaning that competition is still intense. Furthermore, dispersers from accidentally abundant species might more easily colonize because of less competition which stimulates the competitive exclusion process as described before.

Alternatively, we would expect excluding competition (EC) to have a rapid diversity loss. This is, however, not the case under intermediate fragmented conditions (Fig 4D) where species loss is slowest of all competition settings. This unexpected effect can also be attributed to the fact that, under relatively isolated conditions, the high inter-species competition coefficient (1.6) of this competitive setting inhibits the colonization process and thus competitive exclusion process in which accidentally dominant species monopolize the patches. Here a colonizing species experienced strong competition from the founder population which inhibits the competitive exclusion process.

### Coexistence and transient dynamics

We only found stable coexistence in the coexisting competitive setting (CC) (Fig 4A, 4B, and 4C green line), the other competitive settings show transient dynamics resulting in continuous species loss over time until one species survives. Other approaches dealing with competition often found stable coexistence between contrasting strategies. For example, Tilman (1994) [53] found stable coexistence between a superior competitor and a superior disperser, Chesson (2000)[54]found coexistence between species having contrasting sensitivity to environmental fluctuations, while Bolker and Pacala (1998) [55] reported coexistence between species having different colonization ability, exploitation speed of resources and competitive tolerance. The reason that we found nearly no stable coexistence in our results is because we worked with rather similar species having equal colonization, sensitivities to environmental fluctuations (no response diversity), recruitment, mortality and competitive abilities (almost no trait diversity). This choice is in line with the work of Scheffer and van Nes (2006) [56] who reported that evolution selects for many similar species in a single niche. We consider a woodland bird community in competition with each other, the fact that they are competing indicates that there is a niche overlap. Woodland birds often compete for similar food like insects and seeds and nesting opportunities, and respond similarly to food shortage induced by climatic fluctuations, furthermore they generally have good dispersal ability. Nevertheless, we also look at random species assemblies in which we vary recruitment, adult mortality, dispersal and competitive ability of the species. But also here we found limited coexistence under non-fragmented condition (Fig 4A). Our results are in line with the work of Neuhauser and Pacala (1999) [57] who studied effects of stochasticity and explicit space in Lotka-Volterra competition models. They found that stochasticity and explicit space had a negative effect on the coexistence of species in their models. This suggests that deterministic non spatial Lotka-Volterra competitions models allow more coexistence than more realistic spatial explicit and stochastic approaches like we used.

### Neutral theory

We use a metacomunity model of competing species to study the effects of fragmentation on diversity loss. It differs from the neutral models in several ways: we include more realistic spatial relations, competition and Allee effects in our model and we have no speciation process nor a stable species pool. Despite these differences, our main result (the fact that high connectivity reduces biodiversity) was also found in neutral models [27,49]. In standard neutral models individuals of different species use equal space. This, in fact, resembles our neutral competitive setting having the competition index *α*_*ij*_ = 1 for all species interaction. In this competitive setting, the Lotka-Volterra competition as used in our model functions in a similar way to the stochastic recruitment process in neutral models, mortality of adults gives room for replacement by the same or other species affected by immigration. This means that in neutral models the competitive exclusion process, as described in preceding sections, is also present causing species that become accidentally abundant to decrease diversity at high connectivity levels. The neutral models, as far as we know, haven’t been widely adopted by scientists from the field of conservation biology or landscape ecology.

### Implications for conservation

Generally, the proposed ideal landscape configuration for species conservation is based on single-species metapopulation theory [1–6,42]. This framework shows that all species benefit from well-connected landscapes. Our work suggests that, when species interactions are important, a degree of spatial segregation might be a prerequisite for maintaining species richness. This means that metapopulation based landscape evaluation tools may overestimate the value of connectivity and underestimate the value of isolated patches for species conservation. Especially, in cases where species interactions are important metapopulation approaches should be replaced by more subtle meta-community based tools that take effects of species interactions into account. In addition, too much fragmentation may cause metapopulation extinction due to uncompensated local extinction in individual patches in the very long run. If our result are correct, perhaps the best advice for species conservation in fragmented landscapes of north-west Europe and parts of North America, which exist in circumstances where species distributions are shifting [37] and ecosystems are undergoing rapid change [49], would be to aim for spreading of risk by designing heterogeneous landscapes with patches having various levels of fragmentation.

## Supporting Information

S1 File Model details. (ZIP)

S1 Table Hierarchical competitive settings. (DOCX)

S2 Table Results of additional simulations. (DOCX)
