# Correction: Rapid Diversity Loss of Competing Animal Species in Well-Connected Landscapes

**DOI:** 10.1371/journal.pone.0137082

**Published:** 2015-08-26

**Authors:** The PLOS ONE Staff

Fig 5 is incorrect. Please see the corrected Fig 5 here. The publisher apologizes for the error.

**Fig 5:**
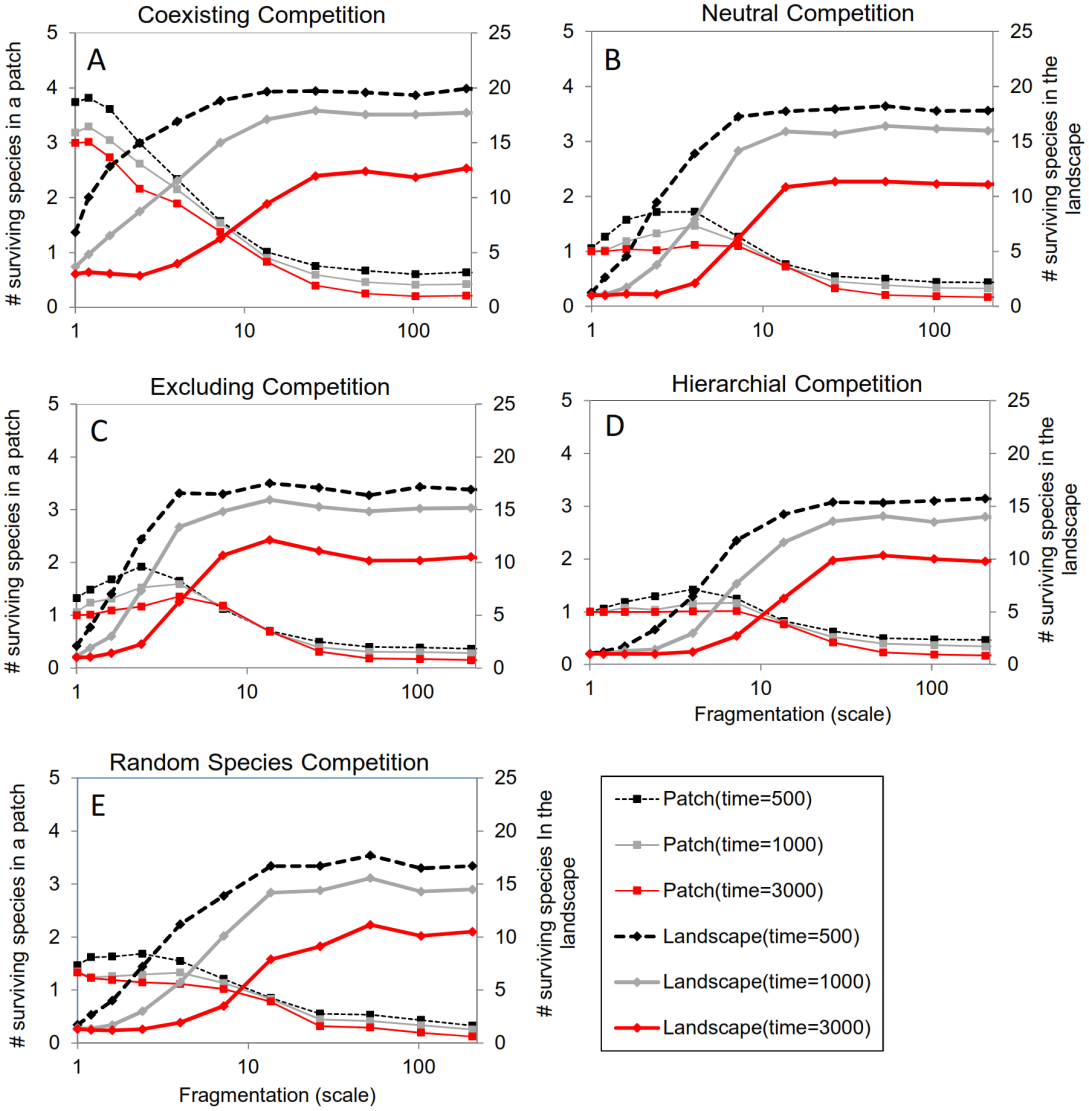
The effect of fragmentation (scale) on the number of surviving species out of 21 under the reference conditions at five competitive settings (A-E). Different lines indicate the amount of surviving species out of 21 at different time intervals (t in years) since the start of the simulation. Bold lines indicate the diversity in the whole landscape and thin lines indicate the average patch diversity. Every point represents the average of 30 simulations. The landscape configuration consists of 100 patches that vary in size and are distributed at random, the average patch carrying capacity is 20 breeding pairs. The figure illustrates that the metapopulation diversity is highest at high fragmentation scales in all competitive settings whereas patch diversity is highest at intermediate fragmentation levels.

In the Introduction, there is an error in the first equation. The asterisk following “F” should be in superscript. Please view the complete, correct equation here:

$$F^{*}=1-\frac{e}{c}\;\text{for }c>e$$ (1)

$$F^{*}=0\text{ for }c<e$$

In the Introduction and Material and Methods, there are errors in equations one through four. The “Ex:” preceding the equations should not be present. Please view the complete, correct equations here:

$$F^{*}=1-\frac{e}{c}\;\text{for }c>e$$ (1)

$$F^{*}=0\text{ for }c<e$$

$$A_{k,i}(t+1)=A_{k,i}(t)+\left(0.5\cdot r_{i}-m_{i}\right)\cdot A_{k,i}(t)\left(1-\frac{\sum_{j=1}^{n}A_{k,j}(t)\cdot \alpha_{ij}}{K_{k}}\right)-E_{k,i}+I_{k,i}$$ (2)

$$P_{k,i}=\text{min}\left(P_{i}(K_{k}),\frac{P_{i}(K_{k})\cdot A_{k,i}(t)}{K_{k}}\right)$$ (3)

$$P_{k\to l}=\frac{2\cdot \text{arcsin}(\text{min}(1,\frac{u_{l}}{d_{k,l}}))}{2\cdot \pi }$$ (4)
