# Supplementary material for: Rapid Diversity Loss of Competing Animal Species in Well-Connected Landscapes
Source: PLoS One. 2015 Jul 28;10(7):e0132383. doi: 10.1371/journal.pone.0132383 (PMC4517897; doi:10.1371/journal.pone.0132383)
Supplement: S1 Table — (DOCX) [file pone.0132383.s002.docx]

**ESM Schippers et al. 2015. Rapid diversity loss of competing animal species in well-connected landscapes.**

S1 Table. The matrix with *α*_ij_ values used in the hierarchical competition scenario, where species 1 is a strongest competitor, species 21 the weakest.
