# Supplementary material for: Rapid Diversity Loss of Competing Animal Species in Well-Connected Landscapes
Source: PLoS One. 2015 Jul 28;10(7):e0132383. doi: 10.1371/journal.pone.0132383 (PMC4517897; doi:10.1371/journal.pone.0132383)
Supplement: S2 Table — (DOCX) [file pone.0132383.s003.docx]

**ESM Schippers et al. 2015. Rapid diversity loss of competing animal species in well-connected landscapes.**

**S2 Table. Species survival in the landscape of aditional simulations**

**Explanation of S2 Table simulations**

Here we describe briefly the changes made to the reference settings and the impact of these changes on the species survival at various fragmentation levels after 1000 years.

*Clustered initial species distribution*,

We initialized the reference simulations with one breeding pair (one male, one female) of each species in each patch meaning that all species are initially present in all patches. To test how this initial setting might affect the outcome of the competition, we initialize the simulations in a contrasting, more clustered way. Hence, we initialized a set of new simulations with 20 breeding pairs of a single species in five patches. So, each patch starts being occupied by a single species.

Results resemble the reference simulations to a large extent (ESM Table S2) only at high isolation levels the diversity is a bit higher due to this different initialization (ESM Table S2).

*Carrying capacity decreased*

When we reduce the carrying capacity of an average patch from 20 breeding pairs to 15 reproductive units per patch we see that the general patterns roughly match that of the reference simulations (ESM Table S2). However, the general diversity level is lower.

*Carrying capacity increased*

When we double the carrying capacity to 40 breeding pairs per patch. The increase of carrying capacity results in higher diversity levels in all the simulations (ESM Table S2) while the diversity is always increasing with isolation. Especially the diversity in the coexisting case at low isolation increased from 3.7 to 8.5 species. However also here the diversity levels are higher at higher fragmentation levels

*Patch size range increased*

In the reference simulations the patch size range is 0.25 - 0.75 km^2^ which corresponds with a carrying capacity of 10-30 breeding pairs. Here, we increase this range to 0.05 - 0.95 km^2^ which corresponds to a carrying capacity of 2 - 38 breeding pairs. The increase of the patch variability results in a small diversity increase in most competitive settings (ESM Table S2).

*Reproduction at low density decreases*

We decrease the maximum recruitment from 1.8 to 1.4 (juveniles per female). This decrease in reproduction causes a strong decrease in the diversity in all the competitive settings. Furthermore there is a marked diversity optimum at intermediate fragmentation levels in all competitive settings (ESM Table S2). However also here the diversity levels are higher at higher fragmentation levels

*Dispersal fraction at carrying capacity decreases*

Here we decrease the dispersal fraction at carrying capacity from 0.6 to 0.3 (year^-1^)

This decrease in the dispersal fraction causes an increase of the diversity in all of the competitive settings. Especially the excluding competitive setting profit from the decrease in dispersal (ESM Table S2). It seems that lowering the dispersal probability lowers the adverse effects species have upon each other. Nevertheless diversity after 100 years was much higher at higher fragmentation levels.

*Dispersal based on multi-species density,*

In the reference simulations the dispersal of individuals is governed by the density of that species in the local patch. Here, we let the density of all species in the patch determine the dispersal of all individuals, so also from other species. We do this because if species experience competition from other species they are likely to respond to their presence. The introduction of this new density dependent dispersal causes a strong decrease of the diversity in all competitive settings (ESM Table S2).

*Each patch has an independent environmental stochasticity,*

In the reference simulations the environmental stochasticity of the recruitment and survival was completely synchronized among species and patches. Here we test the effect of spatial differences in environmental stochasticity for patches and species. No effect of this uncorrelated environmental stochasticity on the results was found (ESM Table S2).

*Each species gets a random competition index*

In our reference simulations, excluding hierarchical competition, we assume equal competition strength between highly similar species but in reality species in a community will have all levels of competitive species interactions. Therefore, we performed a series of simulations with randomly drawn competitive interaction *α_ij_* between 0.4 and 1.6. Meaning that every species has 20 different randomly drawn *α_ij_* which define its competiveness to the 20 other species. For every simulation we completely redraw the competition matrix between species. On average these new simulations match the reference simulations for neutral and excluding competition (ESM Table S2).
